# Pre-operative endoscopic balloon dilatation and its impact on outcome of laparoscopic Heller cardiomyotomy for patients with achalasia: does the frequency and interval matter?

**DOI:** 10.1007/s00464-023-10314-4

**Published:** 2023-07-30

**Authors:** El-Sayed Abou El-Magd, Ahmed Elgeidie, Youssif Elmahdy, Amr Abbas, Mohamed Abdellatif Elyamany, Ibrahem Lotfy Abulazm

**Affiliations:** 1 Department of General Surgery, Faculty of Medicine, Gastrointestinal Surgical Center GISC, Mansoura University, Gehan Street, Mansoura, Al Dakahlia Governorate 35511 Egypt; 2 Faculty of Medicine, Mansoura University, Mansoura, Egypt

**Keywords:** Achalasia, Pre-operative pneumatic balloon dilatation, Outcomes

## Abstract

**Background:**

Many surgeons believe that pre-operative balloon dilatation makes laparoscopic myotomy more difficult in achalasia patients. Herein, we wanted to see if prior pneumatic balloon dilatation led to worse outcomes after laparoscopic myotomy. We also assessed if the frequency of dilatations and the time interval between the last one and the surgical myotomy could affect these outcomes.

**Methods:**

The data of 460 patients was reviewed. They were divided into two groups: the balloon dilation (BD) group (102 patients) and the non-balloon dilatation (non-BD) group (358 patients).

**Results:**

Although pre-operative parameters and surgical experience were comparable between the two groups, the incidence of mucosal perforation, operative time, and intraoperative blood loss significantly increased in the BD group. The same group also showed a significant delay in oral intake and an increased hospitalization period. At a median follow-up of 4 years, the incidence of post-operative reflux increased in the BD group, while patient satisfaction decreased. Patients with multiple previous dilatations showed a significant increase in operative time, blood loss, perforation incidence, hospitalization period, delayed oral intake, and reflux esophogitis compared to single-dilatation patients. When compared to long-interval cases, patients with short intervals had a higher incidence of mucosal perforation and a longer hospitalization period.

**Conclusion:**

Pre-operative balloon dilatation has a significant negative impact on laparoscopic myotomy short and long term outcomes. It is associated with a significant increase in operative time, blood loss, mucosal injury, hospitalization period, and incidence of reflux symptoms. More poor outcomes are encountered in patients with multiple previous dilatations and who have a short time interval between the last dilatation and the myotomy.

Achalasia of the cardia is a rare primary esophageal motility disease, with a rare annual incidence of 1 per 100,000 individuals [1, 2]. That clinical entity is characterized by functional obstruction at the level of the cardioesophageal junction secondary to absent esophageal peristalsis (or esophageal aperistalsis) along with increased lower esophageal pressure [3].

The pathogenesis of achalasia is mediated by the loss of the inhibitory neurons located in the myenteric plexus at the esophageal end. Although the exact etiology remains unknown, it may be the consequence of an autoimmune process secondary to viral infections (measles or herpes) in the presence of genetic susceptibility [4, 5].

Dysphagia is the main symptom of achalasia. Also, patients report regurgitation, weight loss, and chest pain secondary to food stasis. It could also predispose to esophageal cancer [6]. Initial workup for such cases includes esophagogastroduodenoscopy and a barium meal study to exclude patients with pseudoachalasia. Nonetheless, esophageal manometric studies are the mainstay method of diagnosis [7, 8].

Achalasia can be managed by nonsurgical and surgical methods. The former includes balloon dilatation, injection of botulinum toxin, and peroral endoscopic myotomy, whereas the latter includes open or laparoscopic myotomy [9]. Laparoscopic Heller myotomy is the most commonly performed procedure for these cases, as it is associated with the best outcomes and lower recurrence rates. That procedure entails the longitudinal division of the lower esophageal circular muscle fibers proximal and distal to the cardia to relieve that functional obstruction [7].

Despite the previous advantages of the laparoscopic approach, some physicians recommend balloon dilatation for such cases because of its low financial cost and less invasiveness compared to the laparoscopic one [10, 11]. Nevertheless, some patients eventually need surgical intervention when they have insufficient outcomes after balloon dilatation [12].

In addition, there are some concepts believing that endoscopic interventions make subsequent surgical myotomies riskier because of scarring and inflammation of the lower esophageal area [13, 14].

Based on our literature research, the data regarding the effect of previous balloon dilatation on the outcomes of laparoscopic Heller myotomy are scarce. That is why we conducted the current study to elucidate if prior pneumatic balloon dilatation led to worse intraoperative, early, and late postoperative outcomes after the laparoscopic myotomy in such patients.

We also assessed if the frequency of dilatations and the time interval between the last one and the surgical myotomy could affect surgical outcomes.

## Patients and methods

After gaining approval from our University Institutional Review Board (IRB), this retrospective study was conducted (IRB code: R.22.04.1678). The study was designed for adult achalasia patients who underwent laparoscopic Heller myotomy during the period between January 2000 and December 2021.

The data of these patients were retrieved. A total of 460 patients were found eligible to be included. We excluded patients aged less than 18 years, managed via the open approach, diagnosed with recurrent achalasia after prior surgical or laparoscopic myotomy, or who lost at follow-up.

The allocated patients were divided into two groups; the balloon dilatation (BD) group which included 102 patients with a history of previous balloon dilatation whatever its number, and the non-balloon dilatation (non-B) group which did not report such previous intervention (358 patients).

Proper preoperative preparation was done for all patients. History taking focused on medical comorbidities, previous trials of treatment, severity, and frequency of symptoms. The severity of symptoms was classified according to a five-grade scale ranging from 0 to 4 (absent, mild, moderate, severe, and very severe, respectively) [15]. Also, the frequency of symptoms was graded according to the Eckardt score, and the total score was calculated [16].

A barium study was done for all patients to define the shape and degree of esophageal dilatation. The esophageal shape was classified as straight or sigmoid, while its diameter was classified into three categories, less than 3.5 cm, 3.5–6 cm, or more than 6 cm [17].

Other assessments included esophagogastroduodenoscopy (to exclude mechanical obstruction) and esophageal manometry (to confirm the diagnosis). The latter was also used to estimate lower esophageal pressure, relaxing pressure, total length, and intraabdominal length. After routine preoperative laboratory investigations, anesthetic consultation, and signing informed written consent, the laparoscopic procedure was scheduled.

### Laparoscopic Heller cardiomyotomy procedure

The procedure was done under general anesthesia, using the standard five-port laparoscopic technique (supraumbilical camera port, two working ports on each side of the first one, one assisting port at the right anterior axillary line, and one epigastric port for left lobe retraction). The phrenicoesophageal membrane was dissected for exposure of the lower esophagus, and then division of the circular muscle layer was done 6–7 cm on the esophageal side, and 2–3 cm on the gastric side.

The division was done via the laparoscopic dissector (Maryland forceps), ligasure, harmonic device, diathermy over the laparoscopic hook, or a combination of the previous methods. If mucosal injury was encountered, it was repaired by interrupted vicryl 4/0 sutures. The myotomy was then covered by Dor fundoplication, and a surgical drain was inserted under the left lobe, followed by desufflation of the abdomen and port closure.

The operative time, intraoperative blood loss, and surgeon experience were recorded. The latter was classified based on the number of previous procedures performed as follows; less than 5 cases, 5–9 cases, 10–14 cases, or 15–20 cases.

The patients were transferred to the internal surgical ward after the operation. The day of oral intake, duration of hospitalization, and postoperative complications were recorded.

After discharge, follow-up visits were scheduled for all patients, and the maximum duration of follow-up was recorded. During these visits, the Eckardt symptom score was calculated, and the 1-year follow-up reading was recorded. The presence of residual dysphagia and heartburn was recorded. Also, endoscopic assessment was done for heartburn patients to confirm the diagnosis and classify its degree according to the Los Angeles classification [18].

The incidence of failure was also recorded, and it was defined by the need for redo surgery or balloon dilatation after the laparoscopic procedure [19]. Patient satisfaction with the procedure was classified into three-grade Likert scale as follows; very satisfied, satisfied, or unsatisfied.

Our study outcomes included the impact of previous balloon dilatation on intraoperative and postoperative myotomy outcomes. Other objectives included the effect of the number of dilatation trials and the time interval between the last dilatation trial and the laparoscopic procedure on the previous outcomes. Our patients were furtherly subdivided according to the number of previous dilatations (single vs. multiple), and time interval between the last dilatation and surgery [short (< 6 months) and long intervals (6 months or more)].

### Statistical analysis

The statistical analysis was done via SPSS software for MacOs (version 26). Quantitative data were expressed as mean (with standard deviation) or median (with range) according to data distribution. The former data type was compared between the two groups using the Student-*t* test, while the Mann–Whitney test was used for the latter. Categorical data were expressed as numbers and percentages, and compared between the two groups via the Chi-square, Fischer-exact, or Monte-Carlo tests based on the number of categories and the number of cases in each one. For all of the preceding tests, we considered any *p* value less than 0.05 to be statistically significant.

## Results

Patients with previous balloon dilatation had a mean age of 43 years, compared to 41.4 years in the non-BD group. Women represented 60.8% and 53.4% of patients in the BD and non-BD groups, respectively. Their BMI had mean values of 23.23 and 23.76 kg/m^2^ in the same groups, respectively. Both age and gender were statistically comparable between the two groups.

Additionally, the prevalence of smoking and other systemic co-morbidities did not show any statistical differences between the two groups. Most participants in the two groups had ASA class I (76.5% and 77.7% of patients in the two groups, respectively). Other patients had classes II or III, with no significant difference between the two groups.

The duration of symptoms had a median value of 24 months in the two study groups. The score and severity of all symptoms, as well as the total preoperative symptom score, did not differ between the two groups (*p* > 0.05). Pre-operative serum albumin had mean values of 3.9 and 4 g/dl in BD and non-BD groups, respectively (*p* = 0.1).

As regard pre-operative radiological and manometric findings, it was comparable between the two groups. The barium esophagogram revealed comparable esophageal diameter and shape between the two groups. LESP had mean values of 44.06 in BD group versus 44.7 mmHg in the non-BD group (*p* = 0.7). Additionally, total LES length had mean values of 3.7 and 3.7 cm in the two groups, respectively. Table 1 summarizes the previous preoperative data.

**Table 1:** Pre-operative demographic, clinical, laboratory, radiological, and manometric parameters in the two groups

	BD (*n* = 102)	Non-BD (*n* = 358)	*p* value
Age (years)	43.04 ± 12.64	41.46 ± 13.66	0.295
Gender			
Male	40 (39.2%)	167 (46.6%)	0.183
Female	62 (60.8%)	191 (53.4%)	
Smoking	11 (10.8%)	45 (12.6%)	0.627
BMI (kg/m^2^)	23.23 ± 3.75	23.76 ± 3.98	0.235
Co-morbidities			
Chest diseases	11 (10.7%)	31 (8.6%)	0.514
CVD	9 (9.5%)	31 (9.5%)	0.985
Diabetes	6 (5.9%)	13 (3.6%)	0.314
Metabolic diseases	5 (5.4%)	18 (5.6%)	0.944
ASA classification			
ASA 1	78 (76.5%)	278 (77.7%)	0.964
ASA 2	23 (22.5%)	77 (21.5%)	
ASA 3	1 (1%)	3 (0.8%)	
Duration of symptoms	24 (8–120)	24 (4–180)	0.151
Dysphagia score	3 (1–3)	3 (2–3)	0.979
Dysphagia severity			
Mild	4 (3.9%)	16 (4.5%)	0.823
Moderate	65 (63.7%)	216 (60.3%)	
Severe	33 (32.4%)	126 (35.2%)	
Chest pain score	0 (0–2)	0 (0–3)	0.710
Chest pain severity			
0	77 (75.5%)	277 (77.6%)	0.677
1	5 (4.9%)	9 (2.5%)	
2	16 (15.7%)	57 (16%)	
3	4 (3.9%)	14 (3.9%)	
Regurgitation score	1 (0–3)	1 (0–3)	0.367
Regurgitation severity			
0	43 (42.1%)	147 (41%)	0.168
1	9 (8.8%)	54 (15%)	
2	39 (38.2%)	136 (38%)	
3	11 (10.7%)	21 (6%)	
Weight loss	47 (46.1%)	144 (40.2%)	0.155
Weight loss score			
< 5	17 (16.6%)	41 (11.4%)	0.426
5–10	16 (15.7%)	63 (17.6%)	
> 10	14 (13.7%)	40 (11%)	
Total pre-operative symptom score	5 (2–10)	5 (2–11)	0.708
Serum albumin	3.99 ± 0.20	4.02 ± 0.18	0.117
Maximum transverse diameter of esophagus	4 (2–8)	3 (2–8)	0.991
Barium achalasia			
Straight type	74 (72.5%)	286 (79.9%)	0.113
Sigmoid type	28 (27.5%)	72 (20.1%)	
Manometry LESP	44.06 ± 14.11	44.71 ± 12.30	0.713
Total LES length	3.76 ± 0.57	3.74 ± 0.58	0.806
LES abdominal length	2.90 ± 0.57	2.97 ± 0.56	0.338
LES relaxation	64.02 ± 12.10	63.56 ± 14.41	0.815

As shown in Table 2, although surgical expertise did not differ between the two study groups, both operative time and blood loss increased significantly in BD group. The former had mean values of 156.6 and 115.1 min in BD and non-BD groups, respectively (*p* < 0.001). Intra-operative blood loss showed a significant increase in patients in BD group (78.9 vs. 55.6 ml in non-BD groups—*p* = 0.001).

**Table 2:** Intra-operative parameters in both study groups

	BD (*n* = 102)	Non-BD (*n* = 358)	*p* value
Surgical cases experience			
< 5 cases	14 (13.7%)	29 (8.1%)	0.383
< 10 cases	17 (16.7%)	68 (19%)	
< 15 cases	32 (32%)	120 (33.5%)	
< 20 cases	39 (39%)	141 (39.4%)	
Operative time	156.67 ± 38.47	115.14 ± 35.74	< 0.001*
Blood loss mean (SD)	78.9 (61.1)	55.6 (47.3)	0.001*
Muscle thickness			
Thick	44 (43.1%)	173 (48.3%)	0.355
Thin	58 (56.9%)	185 (51.7%)	
Method of dissection			
Harmonic	65 (63.7%)	230 (64.2%)	0.865
Harmonic and diathermy	5 (4.9%)	25 (7%)	
Ligasure	29 (28.4%)	97 (27.1%)	
Ligasure and diathermy	3 (2.9%)	6 (1.7%)	
Accidental mucosal injury	22 (21.6%)	34 (9.5%)	0.001*

Esophageal muscle thickness and the method of dissection did not differ between the two groups. Nonetheless, there was an increased incidence of mucosal perforation in BD group (21.6% vs. 9.5% in the non-BD group—*p* = 0.001).

### Post-operative outcomes

There was a significant delay in oral intake as well as an increase in the duration of hospitalization in association with BD. The former ranged between 1 and 10 days in BD group, while it ranged between 1 and 6 days in non-BD group. The latter ranged between 2 and 20 days in BD group whereas it ranged between 2 and 9 days in non-BD patients. Post-operative symptom scores did not differ between the two groups, as illustrated in Table 3.

**Table 3:** Early postoperative data in the two groups

	Balloon dilatation (*n* = 102)	No balloon dilation (*n* = 358)	*p* value
First day oral	1 (1–10)	1 (1–6)	0.001*
Hospital stay	2 (2–20)	2 (2–9)	0.001*
Post regurgitation score	0 (0–1)	0 (0–1)	0.682
Post dysphagia score	5 (0–1)	0 (0–2)	0.212
Post chest pain score	0 (0–2)	0 (0–2)	0.331
Total symptoms score	1 (0–3)	1 (0–3)	0.573

### Follow-up

At a median follow-up of 4 years, residual dysphagia was reported by 17.6% and 12.3% of patients in BD and non-BD groups, respectively (*p* = 0.162). These patients were considered as “treatment failure”.

There was an increase in the incidence of symptomatic and endoscopic reflux in association with BD group (*p* < 0.001). Patient satisfaction showed a significant improvement in non-BD group, as 77.4% of its patients were very satisfied, compared to only 61.8% of BD group (Table 4).

**Table 4:** Follow-up data in the two study groups

	Balloon dilatation (*n* = 102)	No balloon dilation (*n* = 358)	*p* value
Follow-up period	48 (12–190)	50 (12–192)	0.427
Residual dysphagia	18 (17.6%)	44 (12.3%)	0.162
Heart burn	24 (23.5%)	27 (7.5%)	< 0.001*
Endoscopically GERD	21 (20.6%)	24 (6.7%)	< 0.001*
Endoscopically GERD grades			
Grade A	14 (66.7%)	14 (58.3%)	0.238
Grade B	7 (33.3%)	10 (41.7%)	
Treatment failure	18 (17.6%)	44 (12.3%)	0.162
Satisfaction			
Very satisfied	63 (61.8%)	277 (77.4%)	0.007*
Satisfied	34 (33.3%)	71 (19.8%)	
Unsatisfied	5 (4.9%)	10 (2.8%)	

### Single versus multiple pre-operative BD

When we classified BD group according to the frequency of dilatation (single and multiple BD subgroups), multiple dilatations were associated with a significant increase in operative time, intraoperative blood loss, incidence of mucosal injury, duration of hospitalization, and incidence of clinical and endoscopic reflux esophagitis (Table 5).

**Table 5:** Comparison between the patients with single versus multiple balloon dilatation

Variable	Balloon dilatation		*p* value
	Single (*n* = 59)	Multiple (*n* = 43)	
Operative time mean (SD)	149.5 (40)	169.04 (33.7)	0.01*
Blood loss mean (SD)	58.5 (51.5)	106.98 (62.7)	< 0.001*
Conversion	1 (50)	1 (50)	0.821
Mucosal injury no. (%)	8 (13.5%)	14 (32.5%)	0.02*
Hospital stay mean (SD)	2.8 (2.3)	4.2 (2.8)	0.007*
Total symptom score mean (SD)	2.7 (1.9)	3.7 (2.5)	0.277
Residual dysphagia no. (%)	13 (72.2)	5 (27.8)	0.173
Heart burn no. (%)	9 (37.5)	15 (62.5)	0.02*
Endoscopically GERD no. (%)	7 (31.8)	15 (68.2)	0.005*
Treatment failure no. (%)	13 (72.2)	5 (27.8)	0.173

### Short- and long-interval subgroups

In Table 6, we subdivided patients with previous dilatation according to the time interval between the last dilation and the operation (short- and long-interval subgroups). Patients with short intervals showed a higher incidence of mucosal injury (72.2% vs. 27.3% in the long interval cases—*p* = 0.002) and longer hospitalization periods (4 vs. 2.9 days in the long interval patients—*p* = 0.03). Other parameters showed no significant relationship with the time interval between the dilatation and surgical intervention.

**Table 6:** Comparison between the patients with short versus long time interval between the last dilatation and surgical intervention

Variable	Interval		*p* value
	Short (*n* = 45)	Long (*n* = 57)	
Operative time mean (SD)	165.1 (48.4)	151.9 (28.5)	0.11
Blood loss mean (SD)	90 (72.6)	70.2 (49.2)	0.104
Conversion	2 (100)	–	0.108
Mucosal injury no. (%)	16 (72.2)	6 (27.3)	0.002*
Hospital stay mean (SD)	4 (3.02)	2.9 (2.1)	0.03*
Total symptom score mean (SD)	0.9 (0.8)	1 (0.7)	0.195
Residual dysphagia no. (%)	7 (38.9)	11 (61.1)	0.622
Heart burn no. (%)	13 (54.2)	11 (45.8)	0.257
Endoscopically GERD no. (%)	11 (50)	11 (50)	0.530
Treatment failure no. (%)	7 (38.9)	11 (61.1)	0.622

## Discussion

There is a great deal of controversy among upper gastrointestinal surgeons regarding whether previous endoscopic balloon dilatations would increase the difficulty of the laparoscopic myotomy procedure and impair post-operative outcomes [20].

We conducted the current study to determine a decisive conclusion for that controversy, especially with the large sample included. Initially, one could see almost no significant statistical difference between our preoperative parameters between the two groups, despite the non-randomized nature of the study. That should decrease any bias skewing our findings in favor of one group rather than the other.

Our findings showed a significant increase in mucosal perforation in patients with previous dilatation (21.6% vs. 9.5% in the non-BD group—*p* = 0.001). Previous balloon dilatation leads to minor submucosal hemorrhage which heals by subsequent fibrosis. That fibrosis hinders dissection through the proper surgical planes, making esophageal mucosa more amenable to perforation [21–23].

Beckingham et al. agreed with our findings, as they reported that submucosal fibrosis was present in all their ten patients who underwent laparoscopic myotomy after failed balloon dilatation. That fibrosis made the surgical planes unclear, which increased the difficulty of the procedure and led to the occurrence of mucosal injury in three cases (30%) [24].

Moreover, Morino et al. reported that two patients had intraoperative mucosal perforation out of the seven patients with previous balloon dilatation (28.57%), compared to no perforation cases in the non-balloon dilatation group (*p* < 0.05) [23]. Furthermore, Smith et al. reported a significant increase in intraoperative complications in association with previous endoscopic therapy. These complications included gastric perforation, esophageal perforation, and pneumothorax [25].

Contrarily, Ferguson et al. denied the previous findings, as they did not face any technical difficulties in the dilatation group, and the authors reported that the theory of submucosal fibrosis is unfounded [26].

In our study, we also noticed increased intraoperative blood loss in association with BD (*p* = 0.001). The increased blood loss in BD group could be explained by the improper identification of the correct surgical planes secondary to the fibrosis [24], the increased incidence of mucosal injury, and the increased operative time [27].

We noticed a significant prolongation in the operative time in patients with previous dilatation (156.67 vs. 115.14 min in the non-BD groups—*p* < 0.001). That prolongation in operative time could be explained by the surgical challenges faced in BD group, including challenging dissection, incidence of perforation and requirement of repair. However, Tsuboi et al. contradicted the previous finding, as their operative time was comparable between BD and non-BD groups (178 vs. 171 min, respectively—*p* = 0.498) [28]. Different sample size, surgical expertise, and incidence of complications could explain the previous heterogenicity.

In the current study, there was a significant delay in the start of oral intake as well as a significant increase in the hospitalization period in BD patients, and that could be secondary to the increased incidence of mucosal injury. We often delay oral intake, if mucosal peroration occurred, to the 5th or 6th postoperative day, which could explain the previous differences.

An interesting finding to report was the increased incidence of symptomatic and endoscopic reflux in patients with previous dilatation. That could be explained by the decreased clearance rate of the esophagus in association with previous balloon dilatation, as previously described by Tsuboi et al. [28]. They attributed their findings to the fibrosis secondary to the previous dilatation. Nonetheless, the previous authors did not notice a significant difference in the incidence of that postoperative complication between BD and non-BD groups (*p* = 0.265). We did not find these findings contradictory, as an objective diagnosis of decreased clearance does not necessarily mean that the patient has reflux symptoms.

Portale et al. noticed the increased incidence of heartburn in association with previous dilatation (16% vs. 5.6% in the primary surgery group), despite its insignificance in the statistical analysis [29]. Contrarily, Bloomston et al. reported a comparable incidence of postoperative heartburn between patients who underwent previous balloon dilatation and who did not (24% vs. 20%, respectively) [30].

Although post-operative reflux could be greatly distressing for some patients, it could be a good surgical sign. Smith et al. did not consider reflux a failure after Heller myotomy. Instead, they considered it as a marker for good myotomy, and all of these reflux complaints were successfully managed by medications [25].

In the current study, residual dysphagia was reported by 17.6% and 12.3% of patients in BD and non-BD groups, respectively (*p* = 0.162). In the same context, Tsuboi et al. reported that only one patient had persistent dysphagia in the non-BD group (0.6%) that was managed by balloon dilatation, and no significant difference was noted between the two groups regarding that parameter [28].

Likewise, Souma et al. reported an incidence of 19.2% and 18.1% in the dilatation and non-dilatation groups, respectively (*p* > 0.05) [31]. Another study noted an increased incidence of the same complication in patients with previous balloon dilatation. It was reported in 35% of patients who had previous balloon dilatation versus 0% in patients who did not [30].

We noted no significant difference between the two groups regarding postoperative symptom scores, indicating no significant impact of previous dilatation on postoperative symptom outcomes. Smith et al. also reported comparable dysphagia and regurgitation scores between patients who had previous endoscopic therapy and those who did not [25]. Tsuboi et al. reported similar findings [28].

In our study, the incidence of postoperative failure was comparable between the two groups (17.6% vs. 12.3% in the BD and non-BD groups, respectively—*p* = 0.162). Ferguson et al. reported that preoperative dilatation did not significantly affect postoperative symptoms, whatever the number of dilatation episodes. Good and excellent outcomes were present in 89% and 90% of patients in the BD and non-BD groups, respectively [26].

This coincides with our findings. On the other hand, Smith et al. highlighted the negative impact of previous endoscopic therapy on Heller outcomes, as the incidence of failure was 19.5% in patients with previous endoscopic therapy versus 10.1% in those who did not (*p* < 0.05) [25].

Our findings revealed better patient satisfaction in the non-BD group. The decreased incidence of complications, decreased hospital stay, and decreased incidence of postoperative reflux could explain the previous results.

We noted that the increased frequency of preoperative dilatations is associated with worse outcomes manifested in prolonged operative time, increased blood loss, an increased incidence of mucosal injury, prolonged hospitalization, and an increased incidence of post-operative reflux. The association between multiple previous dilatations and worse outcomes compared to a single-dilatation is difficult to explain. However, the repeated dilatation could induce more fibrosis at the esophageal end, making surgery more challenging. The previous findings should be considered, and such patients should be managed in experienced centers by high-volume surgeons.

Snyder et al. denied any significant impact of the number of previous dilatations on intraoperative parameters, including mucosal perforation (*p* = 0.79), blood loss (*p* = 0.92), and operative time (0.38). Also, the duration of hospitalization was not affected by the dilatation frequency (*p* = 0.15). However, the incidence of failure was significantly higher in the multiple dilatation group (28% vs. 7% in the group with 0 or 1 previous dilatation—*p* < 0.01). Additionally, health-related quality of life improved significantly in the group with 0–1 previous dilatation compared to no improvement in the multiple dilatation group [19].

We also noted a surprising result regarding the impact of the interval between the dilatation and surgical outcomes. It was associated with an increased incidence of mucosal perforation and longer hospitalization periods. Although no previous studies have addressed such an objective, we could explain it by the early edema and mucosal inflammation secondary to the dilatation which could jeopardize surgical planes. It is recommended to delay surgical intervention beyond 6 months in patients with previous dilatations to decrease the risk of mucosal injury and enhance perioperative outcomes.

Although our study included a large sample of patients and addressed the impact of balloon dilatation frequency and the time interval between dilatation and surgery on postoperative outcomes, it has some limitations mainly due to its retrospective nature and sample collection from a single surgical center.

## Conclusion

Previous pneumatic balloon dilatation has a significant negative impact on laparoscopic myotomy outcomes. It is associated with a significant increase in operative time, blood loss, mucosal injury, hospitalization period, delay in oral intake and incidence of reflux symptoms. More poor outcomes are encountered in patients with multiple previous dilatations and who have a short time interval between the last dilatation and laparoscopic myotomy.
